# Sodium-glucose cotransporter 2 inhibitors and constipation: a two-sample mendelian randomization study

**DOI:** 10.3389/fphar.2026.1742232

**Published:** 2026-03-23

**Authors:** Qiuhui Liu, Jing Ning, Zihan Chen, Zihan Wang, Haihua Qian, Dan Zhang, Xi Zhou

**Affiliations:** 1 Department of Preventive Treatment of Disease, Wujin Hospital of Traditional Chinese Medicine, Wujin TCM Hospital Affiliated to Nanjing University of Chinese Medicine, Changzhou, China; 2 School of Integrative Medicine, Nanjing University of Chinese Medicine, Nanjing, China; 3 Department of Anorectal Surgery, Affiliated Hospital of Nanjing University of Chinese Medicine, Jiangsu Province Hospital of Chinese Medicine, Nanjing, China

**Keywords:** circulating metabolites, constipation, mendelian randomization, sodium-glucose cotransporter 2 inhibition, type 2 diabetes mellitus

## Abstract

**Objective:**

Evidence regarding the effect of sodium-glucose cotransporter 2 (SGLT-2) inhibition on constipation is conflicting and the underlying mechanism unknown. We aimed to investigate the causal effect of SGLT-2 inhibition on constipation and the potential mediating role of circulating metabolites.

**Methods:**

We conducted a two-sample Mendelian randomization (MR) study. Genetic instruments for SGLT-2 inhibition were constructed from variants associated with SLC5A2 gene expression and glycated hemoglobin (HbA1c) levels. Summary-level data for constipation and 452 circulating metabolites were obtained from large-scale genome-wide association studies (GWAS). The primary analysis used the inverse-variance weighted method, supplemented by extensive sensitivity analyses. A two-step MR approach was applied to quantify mediation.

**Results:**

Genetically proxied SGLT-2 inhibition was associated with a reduced risk of constipation (OR 0.31, 95% CI 0.19–0.53, *P* < 0.0001). Of the 452 metabolites examined, DSGEGDFXAEGGGVR was significantly associated with both SGLT-2 inhibition and constipation. Mediation analysis indicated that DSGEGDFXAEGGGVR significantly mediated 22.76% of the protective effect.

**Conclusion:**

This study provides genetic support for a causal relationship between SGLT-2 inhibition and reduced constipation risk, and identifies DSGEGDFXAEGGGVR as a potential mediating metabolite. These findings offer insights into a possible pharmacological pathway that may inform future approaches to constipation management.

## Introduction

Sodium-glucose cotransporter 2 (SGLT-2) inhibitors are a class of oral anti-diabetic agents that lower blood glucose by inhibiting renal glucose reabsorption (Heerspink et al., 2016). Beyond their glycemic control, these drugs demonstrate significant benefits for cardiovascular and renal outcomes (Bhatt et al., 2021; Neal et al., 2017; Wiviott et al., 2019; Cannon et al., 2020). However, their effect on gastrointestinal motility, particularly constipation, remains poorly understood and findings are conflicting (Liu et al., 2020; Ishihara et al., 2016; Lu et al., 2016). The underlying pharmacological mechanism is also elusive.

Given their pleiotropic metabolic effects (Cowie and Fisher, 2020; Ferrannini et al., 2016), including potential impacts on circulating metabolites and ion exchangers like the Na+/H+ exchanger (NHE) (Abdulrahman et al., 2022; Baartscheer et al., 2017; Dasari et al., 2023), we hypothesize that SGLT-2 inhibition influences constipation risk by modulating specific circulating metabolites. This hypothesis is supported by prior research indicating structural and functional interactions between SGLT2 inhibitors and sodium-handling proteins (Bisignano et al., 2018; Wright, 2020), as well as evidence of NHE inhibition mitigating fibrosis in other tissues (Arow et al., 2020; Shih et al., 2021).

To empirically test this, we employed a two-sample Mendelian Randomization (MR) design. MR leverages genetic variants as instrumental variables to infer causality (Larsson et al., 2023), and is well-suited to test this hypothesis. This approach is particularly valuable given the growing evidence linking metabolic disturbances, including altered bile acid synthesis and specific amino acid deficits, to constipation pathogenesis (Luo et al., 2023; Yang et al., 2023; Yin et al., 2024; Wang J. et al., 2023). Consequently, we performed a two-step MR analysis to: 1. estimate the causal effect of SGLT-2 inhibition on constipation, and 2. investigate the mediating role of circulating metabolites in this pathway, thereby shedding light on the potential metabolic mechanisms involved.

## Methods

### Study design

**FIGURE 1:**
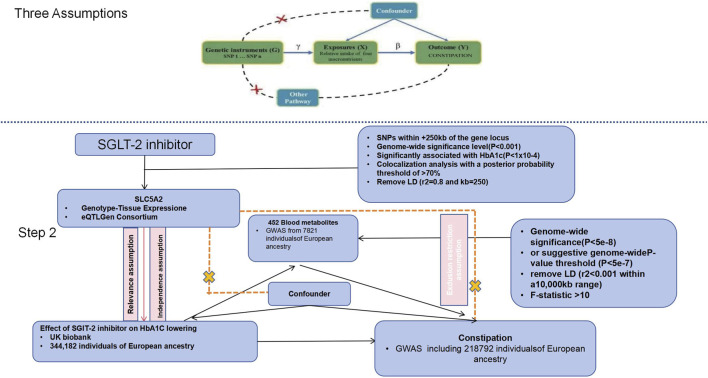
Study design and instrumental variable (IV) selection. The upper panel summarizes the three core MR assumptions: (i) relevance (genetic instruments are associated with the exposure), (ii) independence (instruments are independent of confounders), and (iii) exclusion restriction (instruments influence the outcome only through the exposure). The lower panel illustrates the two-step MR mediation framework with SGLT-2 inhibition as the exposure, blood metabolites as candidate mediators, and constipation as the outcome. Genetic proxies for SGLT-2 inhibition were selected from variants within ±250 kb of the SLC5A2 locus using the criteria shown in the figure (including association thresholds for variant selection, HbA1c association, colocalization posterior probability >70%, and LD pruning settings). Summary statistics were derived from the UK Biobank HbA1c GWAS (N = 344,182, European ancestry), a blood metabolite GWAS of 452 metabolites (N = 7,821, European ancestry), and a constipation GWAS (N = 218,792, European ancestry). Metabolite instruments were selected at genome-wide significance (*p* < 5 × 10^−8^) or suggestive significance (*p* < 5 × 10^−7^), clumped for linkage disequilibrium (LD) (r^2^ < 0.001 within 10,000 kb), and required to have F-statistics >10. SGLT-2, sodium–glucose cotransporter 2; SNP, single-nucleotide polymorphism; LD, linkage disequilibrium; GWAS, genome-wide association study.

This study was carried out with the two-sample MR design (Figure 1). For ensuring that the possible causality was valid, three assumptions must be satisfied in MR analysis (Emdin et al., 2017): 1. genetic variants show robust association with exposure (relevance), 2. genetic variants show independence from confounders (exchangeability), 3. genetic variants affect outcome merely via exposure (exclusion restriction). The present work was reported in line with the Strengthening the Reporting of Observational Studies in Epidemiology Using Mendelian Randomization (STROBE-MR) guidelines (Skrivankova et al., 2021). The work has been reported in line with AMSTAR (Assessing the methodological quality of systematic reviews) Guidelines (Shea et al., 2017) and PRISMA (Preferred Reporting Items for Systematic Reviews and Meta-Analyses) Guidelines (Page et al., 2021).

### Genetic instruments of SGLT-2 inhibitors

Genetic variants of SGLT-2 inhibitors were screened as follows (Figure 1), as mentioned previously (Li et al., 2023). Firstly, genetic loci related to mRNA expression were adopted for creating instrumental variables that represented the therapeutic efficacy of SGLT-2 inhibitors. Later, genetic variants related to SGLT-2 mRNA expression were chosen based on the public datasets of Genotype-Tissue Expression (GTEx) project (Consortium, 2020) as well as eQTLGen consortium (Võsa et al., 2021), then SNPs in ± 250 kb of gene loci, which showed significant relation to relevant traits at the genome-wide significance level, were screened (*P* < 0.001). Thirdly, as SGLT-2 inhibitors can lower glucose levels, the relation between every SGLT-2 variant and glycated hemoglobin (HbA1c, the glucose-lowering effect indicator) level was analyzed, and variants dramatically related to HbA1c were selected (*P* < 1 × 10^−4^). Afterwards, data from genome-wide association studies (GWAS) regarding HbA1c were obtained based on UK Biobank including 344,182 European individuals (Additional file 1: Supplementary Table S1). The exposure and outcome GWAS datasets were obtained from independent consortia; however, due to the use of large biobanks (e.g., UK Biobank) in multiple GWAS, we cannot completely exclude the possibility of partial sample overlap. While MR methods are generally robust to modest sample overlap, we acknowledge this as a potential source of bias.

Finally, co-localization analysis was conducted on SGLT-2 and HbA1c upon the posterior probability threshold >70%. Clumping analysis was carried out using PLINK for removing SNPs from strong linkage disequilibrium (kb = 250 and r^2^ = 0.8) on the basis of 1,000 Genomes Project reference panel of European individuals.

It is important to clarify that the resulting genetic instrument represents lifelong genetic variation in SLC5A2 expression and HbA1c-related pathways, reflecting the cumulative effect of genetically reduced SGLT-2 function from birth. This approach does not directly model pharmacological SGLT-2 inhibition, which may involve acute, dose-dependent, and tissue-specific effects beyond those captured by germline genetic variants. Using expression quantitative trait loci (eQTLs) and glycaemic trait-associated variants as proxies for drug action has inherent limitations, as these variants may influence multiple downstream pathways and cannot fully recapitulate the complex pharmacodynamic and pharmacokinetic profiles of SGLT-2 inhibitors. Consequently, while our findings provide genetic support for a potential causal relationship, they should be interpreted with caution and not be equated with direct evidence of drug efficacy.

### Genetic instruments of circulating metabolites

Data on circulating metabolites were acquired based on Shin et al. (2014). (Additional file 1: Supplementary Table S1). The research explored genetic loci that affected human metabolism, including 7,824 adults in 2 studies on the European population. They reported remarkable associations at the genome-wide level in 145 metabolic loci as well as the biochemical associations with over 400 blood metabolites. Just genetic variants with independent association (LD r^2^ < 0.001 in 10,000 kb) and those with gene-level genome-wide significance (*P* < 5e–8) of every circulating metabolite were analyzed. When SNPs of circulating metabolites were <3, the suggestive genome-wide P-value threshold (*P* < 5e–7) was adopted for identifying the adequate SNPs number (≥3) between circulating metabolites and constipation (Burgess et al., 2013; Wang Q. et al., 2023). For addressing weak instrument bias detected when analyzing instrumental variables, F-statistic was computed for the chosen SNPs to evaluate weak instrument bias. The values >10 were deemed as strong instrument.

### Outcomes

Constipation patients were enrolled into the present work. The summary constipation data were acquired in large-scale GWAS meta-analysis that involved European individuals (Additional file 1: Supplementary Table S1). Ascertainment of constipation was based on outpatient diagnosis.

### Statistical analysis

If just one SNP could be used to construct instrumental variable, MR estimates could be derived by using the Wald ratio approach. If multiple SNPs could be adopted to construct instrumental variable, we adopted the inverse variance-weighted (IVW) algorithm as primary analysis since it offers the most accurate and potent estimates (Burgess et al., 2023). In global test of heterogeneity with IVW, Cochrane’s Q statistic could be applied for evaluating heterogeneity across diverse genetic instruments. As circulating metabolites were strongly associated, multivariable MR was conducted for identifying the probable associated circulating metabolites. In this study, MR-BMA, the two-sample multivariable MR approach capable of identifying true causal risk factors when possible factors are highly associated, was adopted (Zuber et al., 2020). MR analysis was carried out with the weighted linear regression model to combine several circulating metabolites like that in IVW algorithm; subsequently, posterior probabilities regarding the causality of every model were evaluated in the Bayesian framework. Later, posterior probabilities regarding all models including possible circulating metabolites were summed for every possible circulating metabolite, thus calculating marginal inclusion probability (MIP), a value representing the possibility of the causal circulating metabolite for constipation. Besides, model-averaged causal effect estimates (MACE), which represent the mean causality of every circulating metabolite with constipation, were calculated. P-values of every circulating metabolite were computed with permutation approaches. In order to assess the role of circulating metabolites in regulating the relation of SGLT-2 inhibitors with constipation, the two-step MR analysis was carried out for evaluating the regulation of circulating metabolites (Figure 1). Firstly, the role of SGLT-2 inhibitors in 452 circulating metabolites was analyzed by univariable MR analysis (β1). Secondly, circulating metabolites remarkably related to SGLT-2 inhibitors were chosen and their respective influences on constipation (β2) were estimated. Additionally, the mediating proportion of every circulating metabolite in the relation of SGLT-2 inhibitors with constipation was determined by β1 multiplied by β2 and then divided by the overall effect of SGLT-2 inhibitors on constipation.

### Sensitivity analysis

MR-Egger, MRPRESSO, weighted median, simple mode and weighted mode algorithms were adopted for sensitivity analysis. Among them, the MR-Egger algorithm analyzes whether horizontal pleiotropy exists, and the nonzero intercept suggests that possible bias and horizontal pleiotropy exist in IVW estimate (Bowden et al., 2015). Besides, MR-PRESSO algorithm can be used for assessing whether horizontal pleiotropy exists through measuring potential outliers and determining estimates again when all outliers were removed (Verbanck et al., 2018). The weighted median algorithm provides the unbiased causal estimate when over 50% of genetic instruments meet the three MR assumptions (Bowden et al., 2016). As the mode-based approach, the simple mode utilizes causality estimates of individual SNPs for forming clusters and later uses the greatest SNP cluster to estimate causal effect (Hartwig et al., 2017). An identical process is used in the weighted, with assignments of weights to every SNP.

To address multiple hypothesis testing, we applied the Benjamini-Hochberg false discovery rate (FDR) correction (Benjamini et al., 2001). For the sensitivity analyses and assessment of secondary outcomes, a corrected *P*-value < 0.05 was considered statistically significant. Additionally, we computed adjusted *p*-values (q-values) following the Benjamini-Hochberg procedure, with q-values ≤10% considered indicative of significance (Bouras et al., 2022). For the primary analysis evaluating the effect of SGLT-2 inhibition on constipation risk, statistical significance was defined as *P* < 0.05. These complementary approaches allow for a more comprehensive interpretation of causal relationships by accounting for different statistical assumptions and potential biases. All Mendelian randomization analyses were conducted using R software (version 4.3.2) with the TwoSampleMR package (version 0.5.6).

## Results

### Function of SGLT-2 inhibition in constipation

There were altogether 14 SNPs being chosen as genetic instruments of SGLT-2 inhibition, and all of them had the F-statistic >16 (Additional file 1: Supplementary Table S2). SGLT-2 inhibition was significantly related to the decreased constipation incidence (0.31 [0.19–0.53], *P* < 0.0001), and SGLT-2 inhibition led to the reduction of HbA1c level by 1-standard deviation (Table 1). It is worth noting that the observed effect size (OR = 0.31) is relatively large compared with estimates typically reported in drug-proxy Mendelian randomization studies. This may reflect the fact that genetic instruments capture lifelong exposure to reduced SGLT-2 function, which could yield larger cumulative effect estimates than those expected from short-term pharmacological intervention in clinical settings. These findings were robust in sensitivity analyses using MR-PRESSO. No evidence of heterogeneity across instruments was observed (*P* = 0.59), nor was there evidence of horizontal pleiotropy (*P* = 0.26). Leave-one-out sensitivity analysis confirmed the stability of our findings; the IVW estimate remained consistent upon the sequential exclusion of each individual SNP (Supplementary Figures S1–S3), indicating that no single variant disproportionately influenced the overall causal estimate.

**TABLE 1:** MR estimates of the effect of SGLT-2 inhibition on constipation.

Method	nsnp	b	se	pval	or	or_lci95	or_uci95	P-heterogeneity	P-pleiotropy
Inverse variance weighted	14	−1.16	0.27	0.00	0.31	0.19	0.53	0.59	0.26
MR egger	14	0.22	1.21	0.86	1.25	0.12	13.33	0.63	​
Weighted median	14	−1.14	0.38	0.00	0.32	0.15	0.67	​	​
Weighted mode	14	−1.03	0.58	0.10	0.36	0.11	1.11	​	​
Simple mode	14	−1.12	0.67	0.12	0.33	0.09	1.21	​	​

### Impact of circulating metabolites on constipation

**FIGURE 2:**
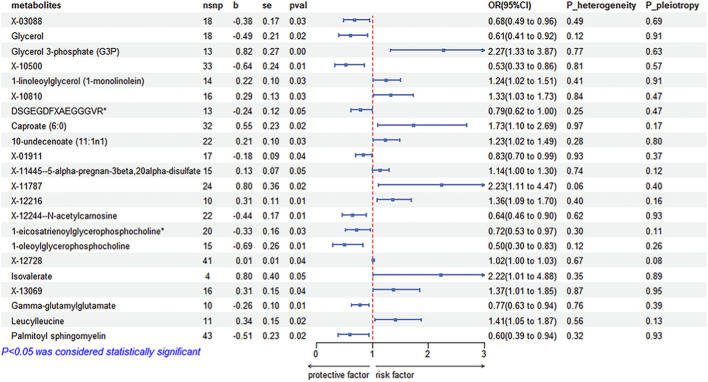
MR estimates of genetically predicted blood metabolites on constipation, showing nominally significant associations. Forest plot displays the causal effect of each metabolite on constipation estimated by the inverse-variance weighted (IVW) MR method. Only metabolites with IVW *p* < 0.05 are shown (non-significant results are not displayed). For each metabolite, the number of instrumental SNPs (nsnp), IVW β, standard error (SE), and *p* value are provided; effect sizes are presented as odds ratios (OR = exp [β]) with 95% confidence intervals (CIs). The red dashed vertical line indicates the null (OR = 1); OR>1 suggests higher constipation risk and OR<1 suggests lower risk per genetically predicted increase in the metabolite. IVW, inverse-variance weighted; SNP, single-nucleotide polymorphism; OR, odds ratio; CI, confidence interval; SE, standard error.

Causal relations of 452 circulating metabolites with constipation were analyzed. We found that 22 circulating metabolites were significantly related to constipation (Additional file 1: Supplementary Table S3; Figure 2). The negative correlations between X-03088, Glycerol, X-10500, DSGEGDFXAEGGGVR, X-12244-N-acetylcarnosine, 1-eicosatrienoylglyrophosphocholine, 1-oleoylglycerophosphocholine, Gamma-glutamylglutamate, Palmitoyl sphingomyelin and constipation were observed (X-03088 (OR = 0.68 [95% CI 0.49–0.96], *P* = 0.03), Glycerol (OR = 0.61 [95% CI 0.41–0.92], *P* = 0.02), X-10500 (OR = 0.53 [95% CI 0.33–0.86], *P* = 0.01), DSGEGDFXAEGGGVR (OR = 0.79 [95% CI 0.62–1.00], *P* = 0.05), X-01911 (OR = 0.83 [95% CI 0.70–0.99], *P* = 0.04), X-12244-N-acetylcarnosine (OR = 0.64 [95% CI 0.46–0.90], *P* = 0.01), 1-eicosatrienoylglyrophosphocholine (OR = 0.72 [95% CI 0.53–0.97], *P* = 0.03), 1-oleoylglycerophosphocholine (OR = 0.50 [95% CI 0.30–0.83], *P* = 0.01), Gamma-glutamylglutamate (OR = 0.77 [95% CI 0.63–0.94], *P* = 0.01), and Palmitoyl sphingomyelin (OR = 0.60 [95% CI 0.39–0.94], *P* = 0.02). Heterogeneity among genetic instruments was not detected, nor was horizontal pleiotropy.

### Function of SGLT-2 inhibition in circulating metabolites

**FIGURE 3:**
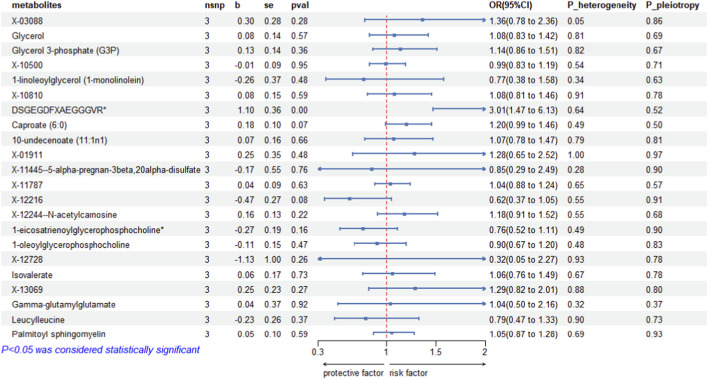
MR estimates of genetically proxied SGLT-2 inhibition on candidate mediator metabolites. Forest plot shows IVW MR estimates for the effect of genetically proxied SGLT-2 inhibition on each candidate mediator metabolite carried forward from the mediation screening step. All candidate metabolites are displayed regardless of statistical significance (including *p* ≥ 0.05). For each metabolite, nsnp, IVW β, SE, and *p* value are reported; effect sizes are shown as exp(β) with 95% CIs to represent the relative change in metabolite levels per unit increase in genetically proxied SGLT-2 inhibition. *P* values for heterogeneity and pleiotropy summarize standard MR sensitivity tests. SGLT-2, sodium–glucose cotransporter 2; IVW, inverse-variance weighted; SNP, single-nucleotide polymorphism; CI, confidence interval; SE, standard error.

The function of SGLT-2 inhibition in 22 circulating metabolites was analyzed, as a result, 1 circulating metabolite showed obvious relations (Additional file 1: Supplementary Table S4; Figure 3). SGLT-2 inhibitors induced DSGEGDFXAEGGGVR expression (OR = 3.01 [95% CI 1.47–6.13], *P* = 0.0028).

### Mediating MR for SGLT-2 inhibition, circulating metabolites, and constipation

**FIGURE 4:**
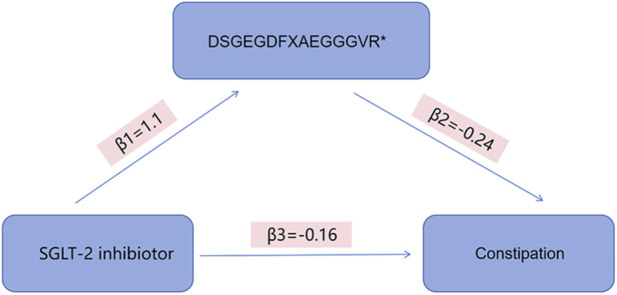
Two-step MR mediation diagram and path coefficients for the metabolite mediator. Path β_1_ denotes the IVW MR estimate for SGLT-2 inhibition → mediator metabolite, and β_2_ denotes the IVW MR estimate for mediator metabolite → constipation. β_3_ represents the direct MR estimate for SGLT-2 inhibition → constipation (direct effect). The MR-based indirect (mediated) effect is calculated as β_1_×β_2_ on the same scale as the MR estimates. SGLT-2, sodium–glucose cotransporter 2; IVW, inverse-variance weighted; MR, Mendelian randomization.

Only one circulating metabolite, DSGEGDFXAEGGGVR, was significantly associated with both genetically proxied SGLT-2 inhibition and constipation risk. Consequently, this metabolite was selected for mediation analysis. Our results indicated that SGLT-2 inhibition exerted an indirect effect on constipation risk through DSGEGDFXAEGGGVR, with a mediation proportion of 22.76% (Figure 4). Due to methodological constraints inherent to two-step Mendelian randomization using summary-level data, the confidence interval for this mediation proportion could not be quantified; therefore, this estimate should be interpreted as preliminary and hypothesis-generating.

## Discussion

### Principal findings

By integrating univariable and multivariable Mendelian randomization with mediation analysis, we provide genetic evidence supporting a pathway wherein genetically proxied SGLT-2 inhibition is associated with a reduced risk of constipation. This protective effect may be partially mediated—to the extent of 22.76%—by the circulating metabolite DSGEGDFXAEGGGVR. These findings offer genetic insights into a potential pharmacological role of SGLT-2 inhibitors and identify a specific metabolite as a potential mechanistic intermediary.

### Interpretation of effect size

It is worth noting that the observed effect size for the association between genetically proxied SGLT-2 inhibition and constipation risk is relatively large compared with estimates typically reported in drug-proxy Mendelian randomization studies. This may reflect the fact that genetic instruments capture lifelong exposure to reduced SGLT-2 function—analogous to lifelong treatment starting from birth—which could yield larger cumulative effect estimates than those expected from short-term pharmacological intervention in clinical settings. Therefore, while our findings provide genetic support for a protective effect, the magnitude of this effect should not be directly extrapolated to predict the short-term efficacy of SGLT-2 inhibitor drugs in routine clinical practice.

### Resolving clinical controversies and positioning the novelty of our study

The relationship between SGLT-2 inhibition and constipation has been a subject of inconsistent clinical reports. While some meta-analyses and trials have suggested a neutral or even adverse effect (Liu et al., 2020; Chen et al., 2019; Kaku et al., 2021), these findings are often constrained by confounding factors in specific patient populations (e.g., diabetes) and the limited statistical power for a secondary outcome like constipation. Beyond constipation, SGLT-2 inhibitors have been implicated in other gastrointestinal outcomes, including rare cases of acute pancreatitis (Li et al., 2025) and potential associations with irritable bowel syndrome (Wang et al., 2025a), highlighting the diverse effects of this drug class on the gut. Our study directly addresses these limitations by employing a genetic paradigm that mimics a randomized trial in the general population. The robust causal evidence we provide helps reconcile these conflicting observations by suggesting that the inherent effect of SGLT-2 inhibition is protective, an effect that may be masked in shorter-term clinical studies or specific disease contexts.

### The DSGEGDFXAEGGGVR-NHE1 axis: a testable mechanistic hypothesis

Moving beyond genetic association, our mediation analysis generates the hypothesis of a potential biological pathway linking SGLT-2 inhibition to constipation risk. We demonstrate that SGLT-2 inhibition elevates levels of DSGEGDFXAEGGGVR, a thrombin-cleaved peptide derived from fibrinogen (de Vries et al., 2017). Crucially, the genetic instrument for this metabolite implicates the SLC9A1 gene, which encodes the sodium-hydrogen exchanger 1 (NHE1). While NHE1 is known to regulate platelet pH and activation (Chang et al., 2015), its expression on the basolateral membrane of colonocytes (Vallés et al., 2015) points to a possible direct role in colonic function. We hypothesize that the DSGEGDFXAEGGGVR-NHE1 axis may represent a component of the protective mechanism. The inhibition of NHE1 activity in the colon could be expected to reduce sodium and concomitant water reabsorption, potentially increasing intestinal water content and softening stools. This proposed “pro-hydration” effect in the colon intriguingly parallels the established diuretic action of SGLT-2 inhibitors in the kidney, suggesting a potentially conserved pharmacological theme. The established importance of water channels like aquaporin-3 in constipation (Ikarashi et al., 2016; Harvey et al., 2008) lends indirect support to the biological plausibility of this water-flux-mediated hypothesis.

While our findings position DSGEGDFXAEGGGVR as a potential mechanistic intermediary, it is important to clarify its role. This fibrinogen cleavage peptide is not specific to constipation pathophysiology. A recent hypothesis-free Mendelian randomization study found that genetically predicted higher levels of DSGEGDFXAEGGGVR were associated with an increased risk of amyotrophic lateral sclerosis (ALS), implicating it in broader neurodegenerative processes (Alhathl et al., 2025). This pleiotropy suggests that DSGEGDFXAEGGGVR is unlikely to serve as a clinically useful biomarker for constipation, as its levels may be influenced by, or contribute to, multiple local and systemic disorders. Instead, our study highlights its value in revealing a specific, testable biological pathway (the proposed DSGEGDFXAEGGGVR-NHE1 axis) that could be a novel pharmacological target for constipation, independent of its role in other conditions.

It is important to emphasize, however, that Mendelian randomization-based mediation analysis does not confirm biochemical pathway activation; therefore, the proposed mechanism involving the DSGEGDFXAEGGGVR-NHE1 axis remains hypothetical and requires experimental validation in appropriate cellular or animal models.

### Potential role of the gut microbiota

Emerging evidence suggests a bidirectional relationship between SGLT-2 inhibition and the gut microbiota. SGLT-2 inhibitors may promote eubiosis by altering the gut environment, potentially increasing the abundance of short-chain fatty acid-producing bacteria (George et al., 2026). Given that gut microbial dysbiosis is a key factor in the pathophysiology of functional constipation (Wang et al., 2025b; Hu et al., 2025; Zheng et al., 2025), it is plausible that the protective effect of SGLT-2 inhibition on constipation risk could be partly mediated through modulation of the gut microbiota. Furthermore, microbiota-derived metabolites can influence host metabolism and disease risk (Zhang et al., 2025). Whether the key metabolite identified in our study, DSGEGDFXAEGGGVR, which is a host-derived fibrinogen peptide, is influenced by gut microbial activity, or if microbial metabolites interact with the proposed NHE1 axis, remains an open question. Future studies integrating metagenomics and metabolomics are warranted to disentangle the complex interplay between SGLT-2 inhibitors, gut microbiota, host metabolism, and colonic function.

## Strengths, limitations, and future directions

The primary strength of this work lies in its MR design, which minimizes confounding and provides robust evidence for causality. To our knowledge, this is the first study to systematically investigate this triad of relationships in the general population. However, several limitations should be acknowledged. First, the genetic proxy represents lifelong variation in SLC5A2 expression and HbA1c-related pathways, which may not fully recapitulate short-term pharmacological SGLT-2 inhibition. The observed effect size (OR = 0.31) should therefore be interpreted with caution, as lifelong genetic exposure may yield larger estimates than clinical drug administration. Second, while our data suggest mediation, the precise mechanism by which SGLT-2 inhibition elevates DSGEGDFXAEGGGVR and how this peptide influences NHE1 require experimental validation in future *in vitro* and *in vivo* studies. Third, our analysis was restricted to European ancestry, limiting generalizability to other populations. Fourth, the absence of an independent replication cohort means our findings require external validation. Fifth, heterogeneity in constipation case definitions across GWAS cohorts may introduce non-differential bias, potentially underestimating true effects. Sixth, the metabolite GWAS had a substantially smaller sample size than the exposure GWAS, potentially reducing statistical power for mediation analysis and increasing weak instrument bias risk. Seventh, we did not perform Steiger filtering to test directionality; although reverse causation is biologically unlikely, we acknowledge this as a limitation. Eighth, due to methodological constraints, confidence intervals for the 22.76% mediation proportion could not be quantified; thus, this estimate should be considered preliminary. Finally, the use of summary-level data precluded adjustment for clinical factors such as constipation duration/severity or treatment duration, which may introduce heterogeneity and influence effect estimates.

## Conclusion

In conclusion, our study provides compelling genetic evidence that SGLT-2 inhibition confers a protective causal effect against constipation. By identifying DSGEGDFXAEGGGVR as a key mediating metabolite and proposing its action through the NHE1 exchanger, we shift the paradigm from mere association to a testable mechanistic model. These insights not only expand the understanding of SGLT-2 inhibitor pleiotropy but also open new avenues for therapeutic strategies in managing gastrointestinal motility disorders.

## Data Availability

The original contributions presented in the study are included in the article/Supplementary Material, further inquiries can be directed to the corresponding author.

## Supplementary material

SUPPLEMENTARY FIGURE 1 Leave-one-out sensitivity analysis for the causal effect of exposure on constipation. Each black point represents the IVW causal estimate excluding that specific SNP, with the corresponding 95% confidence interval. The red point indicates the overall IVW estimate using all SNPs. The analysis demonstrates that no single SNP disproportionately drives the causal effect, supporting the robustness of the primary findings.

SUPPLEMENTARY FIGURE 2 Funnel plot of instrumental variables for the association between exposure and constipation. The plot displays the causal estimates (β) from each SNP against their precision (1/SE). Asymmetry may indicate the presence of directional pleiotropy. The vertical lines represent the pooled estimates from different MR methods: IVW (blue), MR Egger (green), weighted median (orange), and weighted mode (red).

SUPPLEMENTARY FIGURE 3 Scatter plot of SNP effect on exposure versus SNP effect on constipation. Each point corresponds to an individual genetic variant, with error bars representing 95% confidence intervals. The slopes of the lines represent the causal estimates from different MR methods: IVW (blue), weighted median (orange), weighted mode (red), and simple mode (green). The consistency across methods supports the robustness of the causal inference.
