# Supplementary figures and images for: Sodium-glucose cotransporter 2 inhibitors and constipation: a two-sample mendelian randomization study
Source: Front Pharmacol. 2026 Mar 23;17:1742232. doi: 10.3389/fphar.2026.1742232 (PMC13051265; doi:10.3389/fphar.2026.1742232)

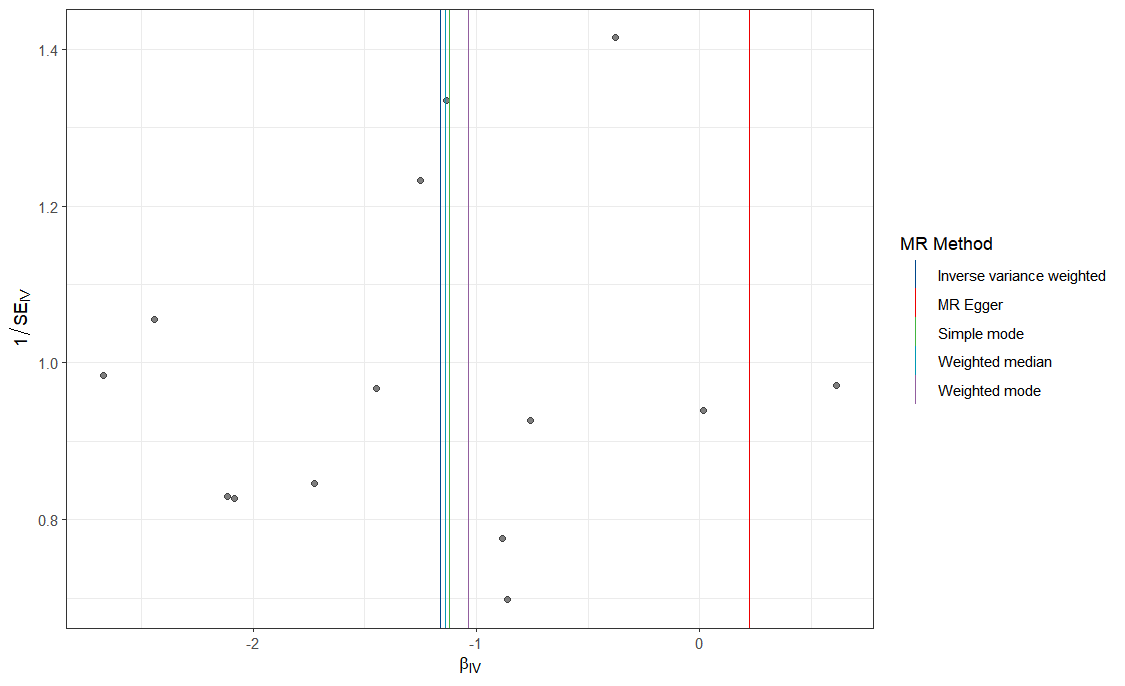

Supplement: Supplementary file 1 [file Image2.png]

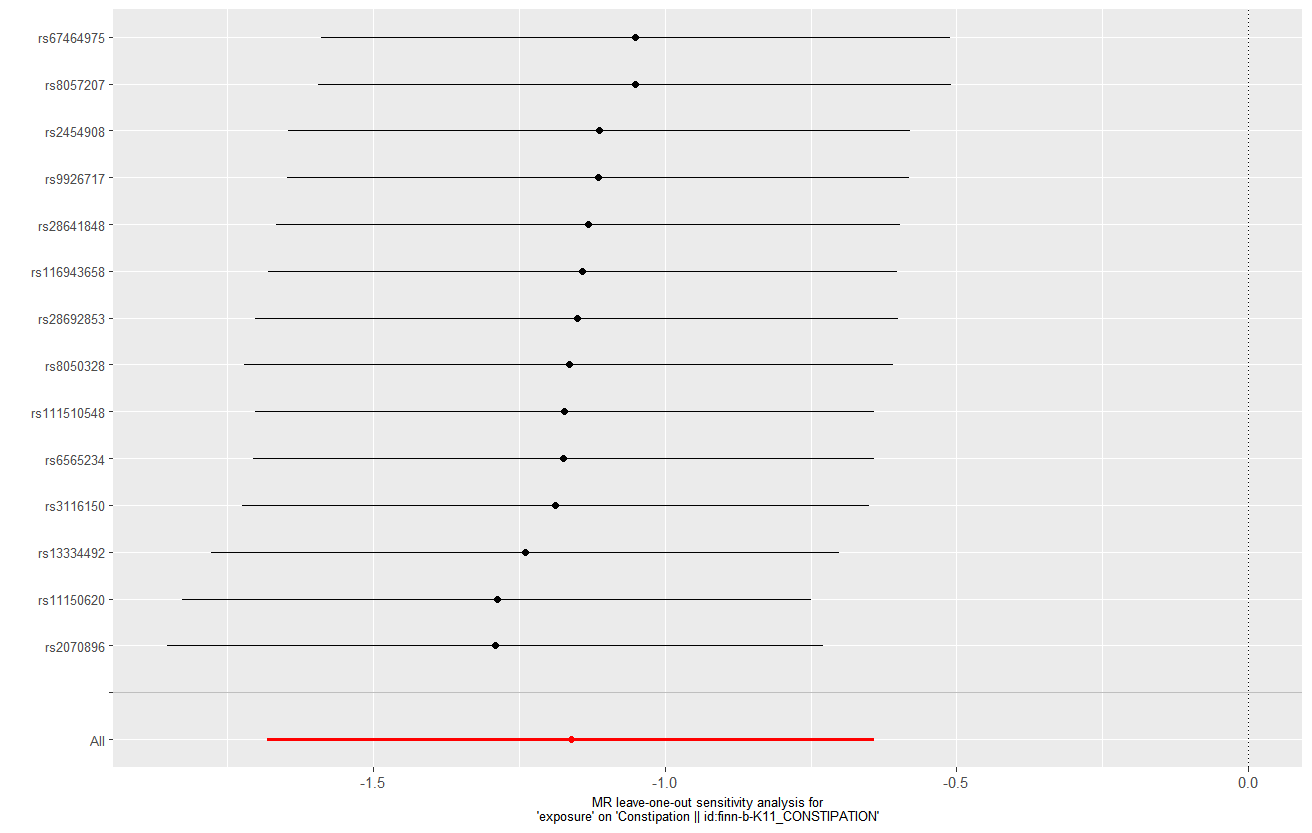

Supplement: Supplementary file 3 [file Image1.png]

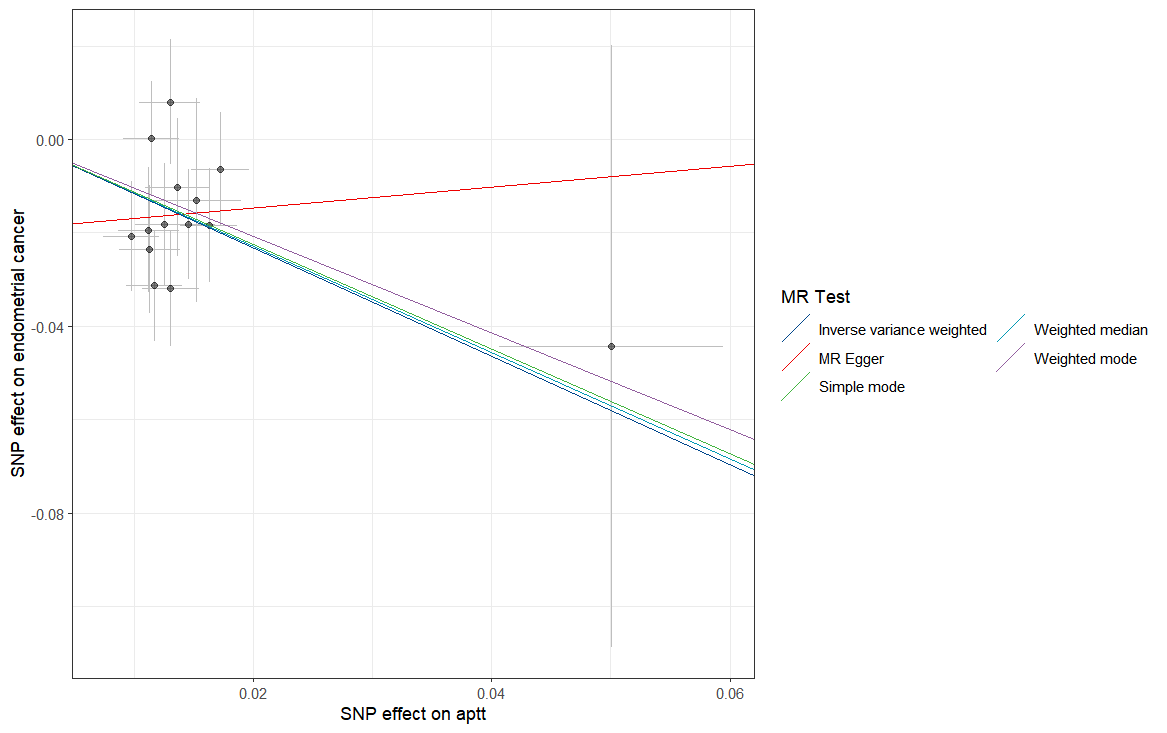

Supplement: Supplementary file 4 [file Image3.png]
